# High-Quality Draft Genome Sequence of Pseudomonas reidholzensis Strain CCOS 865^T^

**DOI:** 10.1128/MRA.01502-18

**Published:** 2019-01-17

**Authors:** Dominik Rutz, David Frasson, Martin Sievers, Jochen Blom, Fabio Rezzonico, Joël F. Pothier, Theo H. M. Smits

**Affiliations:** a Environmental Genomics and Systems Biology Research Group, Institute of Natural Resource Sciences, Zurich University of Applied Sciences (ZHAW), Wädenswil, Switzerland; b Microbiology and Molecular Biology Research Group, Institute of Chemistry and Biotechnology, Zurich University of Applied Sciences (ZHAW), Wädenswil, Switzerland; c Bioinformatics and Systems Biology, Justus-Liebig-Universität, Giessen, Germany; University of California, Riverside

## Abstract

We have sequenced and assembled the genome of Pseudomonas reidholzensis CCOS 865^T^, which was isolated in 2014 from forest soil. Members of the genus Pseudomonas play important roles in environmental systems and are utilized in many biotechnological processes. The genome of this species may provide an important resource for the discovery of novel enzyme activities.

## Abstract (precis)

We have sequenced and assembled the genome of Pseudomonas reidholzensis CCOS 865^T^, which was isolated in 2014 from forest soil. Members of the genus Pseudomonas play important roles in environmental systems and are utilized in many biotechnological processes.

## ANNOUNCEMENT

Bacteria in the genus Pseudomonas are some of the most ecologically important and genetically diverse organisms, and Pseudomonas strains can be isolated from a variety of environmental locations and contexts (1). They are involved in degradation, element cycling, and recycling of biogenic and xenobiotic compounds (2). Based on the enzymatic systems involved in the degradation of aromatic compounds originating from lignin biodegradation (3), pseudomonads have been used in catalytic processes for the biosynthesis of novel fine chemicals (4). Pseudomonas reidholzensis CCOS 865^T^ is a newly identified species within the Pseudomonas putida group and was isolated in 2014 from forest soil in Switzerland (5). Here, we report the draft genome sequence of the potential new biocatalyst Pseudomonas reidholzensis CCOS 865^T^.

Genomic DNA of P. reidholzensis CCOS 865^T^, grown overnight at 28°C in LB medium, was isolated using the NucleoSpin tissue kit (Macherey-Nagel, Düren, Germany) and fragmented using the Covaris E220 ultrasonicator (average target size, 550 bp). Library preparation was performed using the Illumina NeoPrep library system, according to the manufacturer’s instructions. Genome sequencing was performed at the Zurich University of Applied Sciences (ZHAW) using 300-bp paired-end reads and a 550-bp insert library on an Illumina MiSeq instrument (5, 6). For the assembly process, a total of 1,731,754 reads were generated. The SeqMan NGen software 12.1.0 (DNAStar, Madison, WI) was used with standard settings for automatic assembly (5). After further assembly, the final draft genome sequence has a total of 45 contigs, with an *N*_50_ value of 261,911 bp, a length of 6,163,129 bp, and a G+C content of 64.09%. The genome was annotated in GenDB (7), while EDGAR version 2.3 (8) was used for comparative genomics against related pseudomonads.

We confirmed the species delineation against other species of the P. putida group by calculating the genome-to-genome distance (GGDC; version 2.1) values (9) and the average nucleotide identities based on BLAST (ANIb) with JSpeciesWS version 3.0.20 (10). An average ANIb of 82.92% ± 0.37% and GGDC of 28.9% ± 0.8% were obtained with Pseudomonas guariconensis LMG 27394^T^ (GenBank accession number FMYX00000000), Pseudomonas plecoglossicida NBRC 103162^T^ (GenBank accession number BBIV00000000), and Pseudomonas sp. strain GM84 (GenBank accession number AKJC00000000). Based on these results, P. reidholzensis CCOS 865^T^ differs from other members of the P. putida group at the genome level. The first comparative genome analysis of P. reidholzensis CCOS 865^T^ revealed several gene clusters for the degradation of aromatic compounds, such as genes for mandelic acid, vanillin, or gallic acid (11 – 13), which are similar to those found in other Pseudomonas species.

### Data availability.

The draft genome sequence of P. reidholzensis CCOS 865^T^ was deposited at DDBJ/EMBL/GenBank under BioProject number PRJEB28254 and the accession number UNOZ00000000. The version described in this paper is version UNOZ01000000. Raw sequence reads (Illumina) have been deposited at EMBL under the accession number ERR2814816.
